# 2D-^1^H proton magnetic resonance spectroscopic imaging study on brain metabolite alterations in patients with diabetic hypertension

**DOI:** 10.3892/mmr.2015.3305

**Published:** 2015-02-05

**Authors:** ZHEN CAO, BI-DI YE, ZHI-WEI SHEN, XIAO-FANG CHENG, ZHONG-XIAN YANG, YAN-YAN LIU, REN-HUA WU, KUAN GENG, YE-YU XIAO

**Affiliations:** 1 Department of Medical Imaging, The Second Affiliated Hospital, Shantou University Medical College, Shantou, Guangdong 515041, P.R. China; 2 Department of Medical Imaging, The Central Hospital of Huizhou City, Huizhou, Guangdong 516001, P.R. China

**Keywords:** cerebral metabolism, diabetic hypertension, magnetic resonance spectroscopy

## Abstract

The aim of the present study was to investigate the possible metabolic alterations in the frontal cortex and parietal white matter in patients with diabetic hypertension (DHT) using proton magnetic resonance (MR) spectroscopic imaging. A total of 33 DHT patients and 30 healthy control subjects aged between 45 and 75 were included in the present study. All subjects were right-handed. The spectroscopy data were collected using a GE Healthcare 1.5T MR scanner. The multi-voxels were located in the semioval center (repetition time/echo time=1,500 ms/35 ms). The area of interest was 8×10×2 cm in volume and contained the two sides of the frontal cortex and the parietal white matter. The spectra data were processed using SAGE software. The ratios of brain metabolite concentrations, particularly for N-acetylaspartate (NAA)/creatine (Cr) and Choline (Cho)/Cr were calculated and analyzed. Statistical analyses were performed using SPSS 17.0. The NAA/Cr ratio of the bilateral prefrontal cortex of the DHT group was significantly lower than that of the control group (left t=−7.854, P=0.000 and right t= −5.787, P=0.000), The Cho/Cr ratio was also much lower than the control group (left t=2.422, P=0.024 and right t=2.920, P=0.007). NAA/Cr ratio of the left parietal white matter of the DHT group was extremely lower than that of the control group (t=−4.199, P=0.000). Therefore, DHT may result in metabolic disorders in the frontal cortex and parietal white matter but the metabolic alterations are different in various regions of the brain. The alteration in cerebral metabolism is associated with diabetes and hypertension. The ratios of NAA/Cr and Cho/Cr are potential metabolic markers for the brain damage induced by DHT.

## Introduction

Diabetes and hypertension are common metabolic diseases. Previous epidemiological studies have indicated that the two diseases often occur one after the other and cause a condition termed diabetic hypertension (DHT). According to a World Health Organization study, ~60% of patients with type 2 diabetes also suffer from hypertension (1). When a patient suffers the two diseases at the same time, damage to target organs, such as the brain, is more significant.

Brain damage is a serious chronic complication of DHT. Previous studies have demonstrated that following cerebral infarction, cerebral white matter lesions and other medical imaging changes are observed (2,3). These changes may be examined using conventional tools, including computed tomography (CT) and magnetic resonance imaging (MRI); however, such tools can only provide the morphological information on the late complications of brain damage caused by DHT. For a number of diseases, it is crucial and useful for diagnosis and treatment (4,5) to know at an early stage the metabolic information of the organs affected by the disease, as the metabolic changes usually occur prior to the pathological alterations in the tissue structure.

Magnetic resonance spectroscopy (MRS) is a safe, non-invasive means of performing biochemical analyses *in vivo*. Using this technique, information regarding the metabolic alterations in different tissue types, including brain tissues (6) can be observed. Generally, MRS consists of two techniques, termed single voxel spectroscopy (SVS) and multi-voxel spectroscopy (MVS). SVS receives a signal of a volume limited to a single voxel. The advantages of SVS include a shorter acquisition time, more explicit spatial localization, more homogeneous shimming and improved water suppression. However, it has a number of crucial disadvantages. Notably, only one spectrum may be obtained from one data acquisition and the comparison cannot be performed simultaneously between the contralateral tissues during one data collection. Furthermore, the accuracy of SVS is also affected by the localization of voxel. MVS, also known as chemical shift imaging, is a multi-voxel technique, which allows for the measurement of larger volumes of tissue that can be divided into smaller voxels during the processing period. It is capable of comparing spectra from multiple brain regions simultaneously. When the lesion is uneven in quality, the use of small voxels may reduce the average volume effect (7,8).

Numerous studies have predominantly used SVS to investigate the brain metabolites in patients with diabetes or hypertension. It was identified that the N-acetylaspartate (NAA) concentrations in the bilateral hippocampus were lower in diabetic patients than in the normal group and the Choline (Cho) concentrations in the bilateral hippocampus were higher in the normal group (9). There are also certain studies, which noted that when comparing type 2 diabetic patients with the control group, the NAA/creatine (Cr) and Cho/Cr values were decreased in the frontal cortex (10–12). In addition, Catani *et al* (13) observed that the NAA/Cr ratio and Cho/Cr values of the white matter were not altered for patients in the hypertension group and those in the control group. Ben Salem *et al* (14) found that the NAA/Cr values of bilateral thalamus and the insular cortex were lower in hypertensive patients than in the normal control group. Previous studies (15-18) indicated that cerebral blood flow with hypertension usually induced early changes in the white matter of bilateral frontal cortex and the parietal lobe. In addition, lacunar infarction often occurred in the basal ganglia, corona radiate and centrum ovale in patients with diabetes.

Currently, there are few studies that focus on the metabolic changes in the brain of patients with DHT. Thus, in the present study, the technique of MVS was used and a semioval center was selected as the region of interest for monitoring the early brain metabolite changes in DHT.

## Materials and methods

### Subjects

A total of 33 patients with DHT (14 males and 19 females; mean age, 62.8±8.6 years) were enrolled in the present study. A total of 30 age-matched volunteers (20 males, 10 females, mean age 59.8±7.7 years) were included as the control group. All subjects were right-handed.

All patients diagnosed with DTH had to meet the following criteria: The patient had a fasting plasma glucose level ≥7.0 mmol/l, random blood glucose level ≥11.1 mmol/l or following an oral glucose tolerance test (OGTT) a blood glucose level ≥11.1 mmol/l after 2 h; they were not taking antihypertensive drugs and they had a systolic blood pressure (SBP) ≥140 mmHg and (or) diastolic blood pressure (DPB) ≥90 mmHg. For the control group, all selected candidates had to meet the following criteria: They were not taking anti-hypertensive drugs; they had an SBP <140 mmHg and a diastolic blood pressure <90 mmHg; their fasting blood glucose level was <6.1 mmol/l and following OGTT, they had a blood glucose level of <7.8 mmol/l after 2 h, and no history of abnormal lipid metabolism.

Exclusion criteria for the present study included patients who had previously suffered central nervous system damage caused by other diseases or a similar central nervous illness, history of drug dependence or other substance abuse, a history of mental illness or a history of severe medical illness.

### Proton magnetic resonance spectroscopy (2D-^1^HMRS) imaging

The present study was conducted in the Medical Imaging Department of The Second Affiliated Hospital of Shantou University Medical College (Shantou, China) between December 2011 and July 2012. The study was approved by the ethics committee of Shantou University Medical College (The Second Affiliated Hospital, Shantou, China)The local ethics committee approved the study and all volunteers provided informed consent.

**Figure 1:**
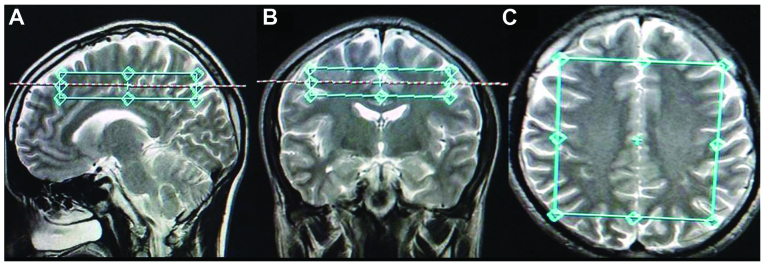
Three plane location map of volume of interest for proton magnetic resonance spectroscopy. Volume of interest located in (A) sagittal position, (B) coronal position and (C) axial position

The MR study was performed on a 1.5T GE Signa HDX scanner (GE Healthcare, Wauwatosa, WI, USA) with an 8-channel head coil. During the scan, the heads of subjects were fixed with a sponge pad in order to enable the patient to remain stationary. Routine MRI imaging, including an axial T_1_-weighted image (WI), a T_2_WI and a sagittal or coronal T_2_WI was conducted for each subject. The scanning parameters were as follows: T_1_WI [repetition time/echo time (TR/TE)=2,162 ms/20.6 ms, matrix=320×256, field of view (FOV)=24×18 cm, slice thickness=7 mm, gap=1.5 mm]; T_2_WI (TR/TE=4,420 ms/112.1 ms, matrix=384×256, FOV=24×18 cm, slice thickness=7 mm, gap=1.5 mm). The scan range was from the parietal to the foramen magnum. Localized proton spectra were acquired using the point resolved selective spectroscopy sequence. The parameters were as follows: TR=1,500 ms, TE=35 ms, matrix=512×512, phase and frequency=18×18, FOV=16×16 cm, number of excitations=1.0. A total of three planes were used to determine the volumes of interest (VOI) and the VOI was placed in the central layer of a semioval. The volume of VOI was 7×10×2 cm and each single small voxel size was 1.58 cm^3^ (Fig. 1). In order to meet the requirements for analyzing the metabolites, the full width at half maximum was controlled within 10 Hz and the inhibited water level was >98%, which can be accomplished by alternatively using the automatic and manual shimming methods. The total acquisition time for MRS data was 8 min and 12 sec.

### Data processing

**Figure 2:**
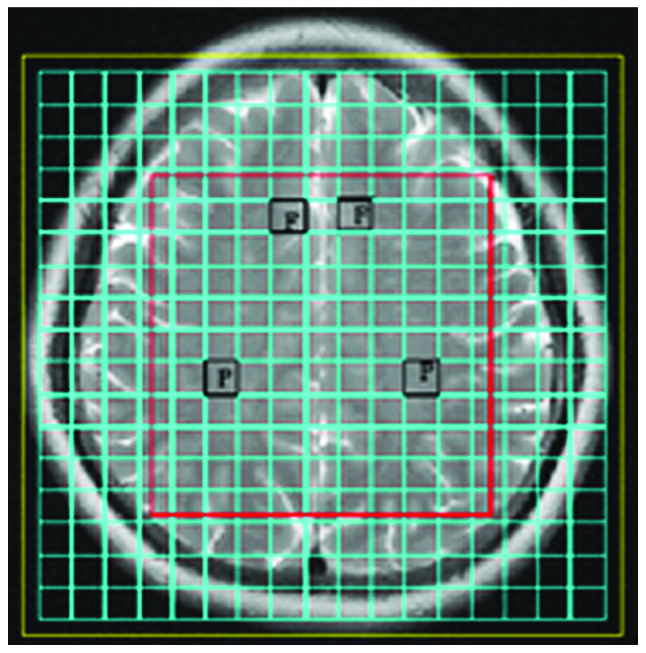
Regions of interest of magnetic resonance spectroscopy in the bilateral frontal cortex and the parietal white matter.

Following the 2D-^1^HMRS scan, the data were imported, and SAGE, version 2007.1 (GE Company, Waukesha, WI, USA) and LCModel software, version 6.3 (http://s-provencher.com/pages/lcmodel.shtml) were applied to signal averaging, baseline correction, phase cycling, metabolite identification and calculating the ratio of the metabolites in each voxel. In the present study, the bilateral prefrontal cortex and bilateral parietal white matter were selected as regions of interest (Fig. 2) and the ratios of NAA/Cr and Cho/Cr were calculated.

### Statistical analysis

The data were analyzed using SPSS 17.0 (SPSS, Inc, Chicago, IL, USA). An independent-sample t-test was used to compare the metabolic ratios in different groups. P<0.05 was considered to indicate a statistically significant difference. The measurement data were expressed as the mean ± standard deviation.

## Results

**Figure 3:**
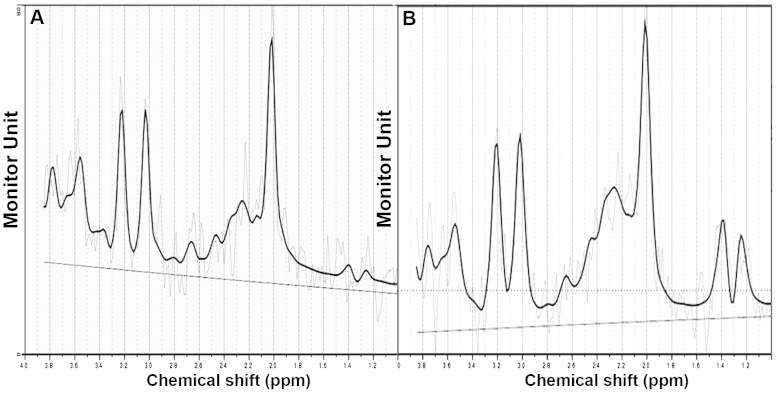
Magnetic resonance spectrum obtained from the left frontal cortex. (A) Diabetic hypertension group and (B) control group. Thin gray line, original spectra; thick black line, fitting spectra from LCModel software; bottom line, base line of spectra.

**Figure 4:**
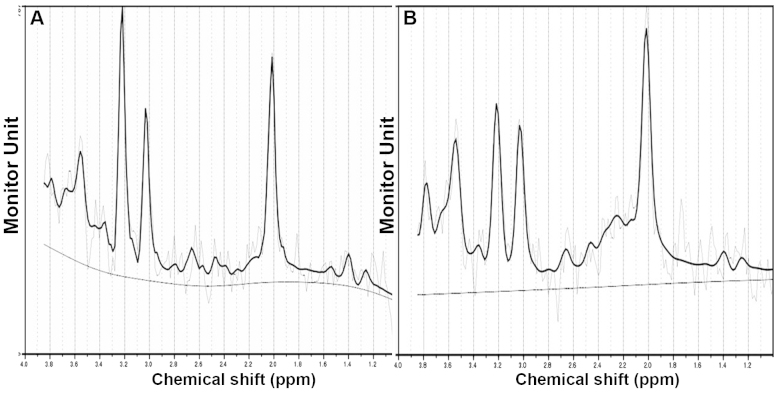
Magnetic resonance spectrum obtained from the right frontal cortex. (A) Diabetic hypertension group and (B) control group. Thin gray line, original spectra; thick black line, fitting spectra from LCModel software; bottom line, base line of spectra.

The age and gender differences did not reach statistical significance between the DHT and the control group. High quality 2D-^1^HMRS data were acquired. The metabolite peak intensities were as follows: NAA=2.02 ppm, Cr=3.0 ppm, Cho=3.2 ppm and MI=3.56 ppm. The ratios of NAA/Cr and Cho/Cr of the bilateral prefrontal cortex and the parietal lobe white matter in the two groups are shown in Tables I and II. The NAA/Cr ratios in the bilateral prefrontal cortex of the DHT group were significantly lower than that of the control group (left t=−7.854, P=0.000; right t=−5.787, P=0.000), The Cho/Cr ratios in bilateral prefrontal cortex in the DHT group were higher than that of the control group and revealed a significant difference (left t=2.422, P=0.024 and right t=2.920, P=0.007; Figs. 3 and 4).

**Figure 5:**
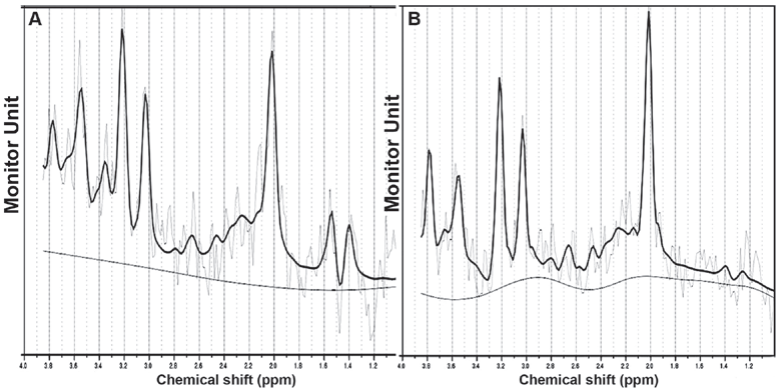
Magnetic resonance spectrum obtained from the left parietal white matter. (A) Diabetic hypertension group and (B) control group. Thin gray line, original spectra; thick black line, fitting spectra from LCModel software; bottom line, base line of spectra.

**Figure 6:**
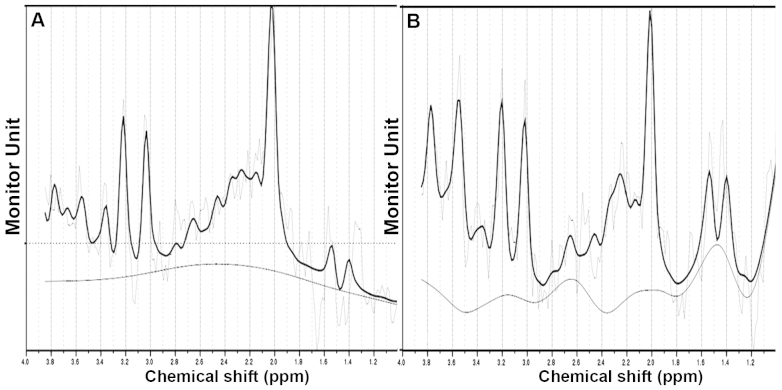
Magnetic resonance data obtained from the right parietal white matter. (A) Diabetic hypertension group and (B) control group. Thin gray line, original spectra; thick black line, fitting spectra from LCModel software; bottom line, base line of spectra.

Compared with the control group, the NAA/Cr levels in the left parietal lobe white matter in the DHT group decreased and were significantly different (t=−4.199, P=0.000). There was a downward trend in the NAA/Cr ratio in the right parietal white matter in the DHT group, however it was not statistically significant (t=−1.215, P=0.229). No statistically significant differences were identified in the Cho/Cr ratio in the bilateral parietal lobe white matter between the DHT group and the control group (left t=0.874, P=0.386; right t=−0.432, P=0.667; Figs. 5 and 6).

## Discussion

DHT has a high incidence and may increase the extent of brain damage associated with diabetes and simple essential hypertension as patients have numerous coinciding risk factors (19). The pathogenic mechanism remains to be elucidated; however, it has been reported to be closely associated with cerebrovascular damage, oxidative stress and non-enzymatic protein glycosylation. These pathophysiological changes may be identified by observing the relevant metabolite alterations (20,21).

MRS, which is a non-invasive imaging technique with reproducibility is a method used to detect metabolites in the body (22). MRS can currently detect numerous types of metabolites, including NAA, Cho, Cr, lactate, lipids, alanine, glutamic acid, γ-amino butyric acid, inositol and a number of others. Cr is used as a reference standard with other metabolites as it is not usually affected by pathological states (23). Therefore, the ratios of NAA/Cr and Cho/Cr to a certain extent may reflect the NAA and Cho concentration alterations.

The levels of the amino acid NAA in the brain are only marginally lower than that of glutamic acid, which is the most abundant amino acid in the brain. NAA is primarily found in neuronal cell bodies and axons, but not in glial cells, therefore NAA is considered to be a marker for neurons. It is generally accepted that a decrease in NAA reflects a loss of neurons, a decline in neuronal activity, a metabolic decrease in the gray matter and also axonal injury in the white matter (24,25). Although Kario *et al* (26) identified that NAA had an independent association with type 2 diabetes, a study has also revealed that diabetes may cause DNA damage in hippocampal neurons, leading to the reduction in neurons and further affecting the synaptic plasticity (27). In addition, the studies by Kreis *et al* (28) and Kreis and Ross (29) indicated that NAA levels were significantly decreased in the parietal white matter of patients with diabetes. In addition, a study by Shiino *et al* (30) demonstrated that the NAA/Cr ratio was significantly lower in the lesions of subcortical arteriosclerotic encephalopathy compared with that in normal tissue. The authors hypothesized that the NAA level was associated with the ongoing hypertension, which led to chronic cerebral circulatory disorders, ischemia, hypoxic changes and neuronal loss. In the present study, The NAA/Cr levels in the bilateral prefrontal cortex were observed to be lower in the DHT group than those in the control group and the difference was statistically significant. The reason for these findings may be that the prefrontal cortex is sensitive to ischemia. It is well-established that hypertension and diabetes can cause vascular wall thickening and luminal stenosis, which decreases cerebral tissue perfusion and results in ischemia and hypoxia in the brain. Under the pathological state of sustained ischemia, the NAA level decreases due to neuronal cell and axonal loss (31). In the present study, it was also identified that the NAA/Cr ratios in the DHT group were lower in the left parietal white matter than those in the control group and the difference was statistically significant. The main reason for these alterations is that the left hemisphere is usually dominant in right-handed subjects, thus in these individuals it has a lower tolerance to ischemia than the right side and is more vulnerable to neuronal damage and metabolic disorders (32).

The Cho level is higher in glial cells than in other types of cells, thus it is classified as a metabolic biomarker of glial cells (33). Cho is a constituent of acetylcholine and phosphatidylcholine, and it is also a constituent of cell membranes (34). A rise in the Cho level indicates intense metabolic activation of the cell or membranous disintegration. The reduction in Cho may be relevant to the renovation of the membrane phospholipids, inositol synthesis, cell density changes, endocrine state and local metabolic rate changes (35,36). In addition, the Cho level also reflects the severity of inflammation, with a Cho increase indicating a greater level of inflammation.

In the present study, the Cho/Cr values of patients with DHT in bilateral prefrontal cortex were found to be increased compared with those in the normal control group. This result may be associated with the interaction of diabetes and hypertension. These two diseases damage the brain blood vessels so that ischemia/hypoxia is induced in the gray matter. Ultimately, the nerve cells of the gray matter degenerate and undergo necrosis, resulting in disintegration of the nerve cell membranes. It may also be relevant that there is increased inflammation in DTH. Liu *et al* (37) suggested that type 2 diabetes is an inflammatory disease and thus the increased inflammatory mediators may result in raised Cho/Cr levels (38,39).

To conclude, based on the present experimental results and compared with the existing data, it was identified that DHT may result in metabolic disorders in the frontal cortex and parietal white matter; however, the metabolic alterations are distinct in these regions of the brain. Therefore, the ratios of NAA/Cr and Cho/Cr, which can be detected by 2D-^1^HMRS in these regions, may be used as potential metabolite markers for the brain damage induced by DHT.

## Figures and Tables

**Table I:** Magnetic resonance spectroscopy values of the diabetic hypertension group and the control group in the prefrontal cortex.

Variable	NAA/Cr		Cho/Cr	
	Left	Right	Left	Right
DHT	0.707±0.257	1.071±0.298	0.396±0.086	0.441±0.114
CON	1.483±0.499	1.506±0.298	0.311±0.091	0.334±0.068
t-value^a^	−7.854	−5.787	2.422	2.920
P-value	0.000	0.000	0.024	0.007

a Independent samples t test. NAA, N-acetylaspartate; Cr, creatine; Cho, choline; DHT, diabetic hypertension; Con, control.

**Table II:** Magnetic resonance spectroscopy values of the diabetic hypertension and the control group in the parietal white matter.

Variable	NAA/Cr		Cho/Cr	
	Left	Right	Left	Right
DHT	1.020±0.512	1.316±0.373	0.349±0.112	0.315±0.088
CON	1.500±0.379	1.450±0.503	0.327±0.086	0.324±0.085
t value^a^	−4.199	−1.215	0.874	−0.432
P value	0.000	0.229	0.386	0.667

a Independent samples t test. NAA, N-acetylaspartate; Cr, creatine; Cho, choline; DHT, diabetic hypertension; Con, control.
